# Comparative High Voltage Impulse Measurement

**DOI:** 10.6028/jres.101.063

**Published:** 1996

**Authors:** Gerald J. FitzPatrick, Edward F. Kelley

**Affiliations:** National Institute of Standards and Technology, Gaithersburg, MD 20899-0001

**Keywords:** high-voltage impulse, high-voltage reference measurement systems, impulse measurements, standard lightning impulse, transient measurements

## Abstract

A facility has been developed for the determination of the ratio of pulse high voltage dividers over the range from 10 kV to 300 kV using comparative techniques with Kerr electro-optic voltage measurement systems and reference resistive voltage dividers. Pulse voltage ratios of test dividers can be determined with relative expanded uncertainties of 0.4 % (coverage factor *k* = 2 and thus a two standard deviation estimate) or less using the complementary resistive divider/Kerr cell reference systems. This paper describes the facility and specialized procedures used at NIST for the determination of test voltage divider ratios through comparative techniques. The error sources and special considerations in the construction and use of reference voltage dividers to minimize errors are discussed, and estimates of the measurement uncertainties are presented.

## 1. Introduction

Accurate high voltage measurements are required by the electric power industry for instrumentation, metering, and testing applications [1, 2]. Similarly, there is a need for accurate measurements of high voltages in pulsed power machines to monitor and optimize machine operation [3]. The accurate measurement of fast transient voltages is also important in the assessment of their effects on electrical power equipment and insulation in order to improve system reliability. Additionally, for the correct evaluation of transient voltage effects on apparatus or dielectrics, the peak voltage and waveshape must be accurately known.

Steady-state high voltages can be measured with much smaller uncertainties than high-voltage transients can be. For example, calibrations of dc high-voltage dividers for divider ratio have been routinely performed in the range of 10 kV to 100 kV with relative uncertainties of less than 0.01 % [4]. AC divider ratios have been calibrated over the same voltage range [4] with 0.05 % relative uncertainties. High-voltage impulses, on the other hand, are much more difficult to measure accurately because of the wide-bandwidth devices and instrumentation necessary to faithfully capture the high-frequency components of these transient signals. Typically, the measurement devices used for scaling the voltages to measurable levels must be physically large to be capable of withstanding the high voltages imposed on them and their large size makes them susceptible to wave propagation effects, pickup of extraneous signals, stray capacitance, and residual inductance effects that distort the measurements of fast transients. Recently, international standards on high-voltage test techniques have been introduced that require voltage dividers used in high-voltage impulse measurements to be traceable to national standards [1, 5]. A facility for the testing of pulse voltage dividers has been developed in response to the needs of the electric power and the pulsed power communities. The facility consists of a set of Kerr electro-optic measurement systems having overlapping voltage ranges and a reference voltage divider. The Kerr systems are well-suited for impulse voltage measurements because of their excellent high-frequency response characteristics and relative immunity from electromagnetic interference. The reference voltage dividers developed at NIST are physically small, oil-immersed devices with response times of the order of 10^−9^ s. The measurement systems described in this paper are designed for testing compact resistive high-voltage dividers of the type used in pulse power machines, but the techniques are applicable to the testing of free-standing impulse voltage dividers used by the electric power industry.

This paper describes techniques developed at NIST to reduce the measurement uncertainties in high-voltage impulse measurements made with two types of high-voltage devices: resistive high-voltage dividers and electro-optic Kerr cells. These two types of systems are based on very different measurement principles. The voltage divider samples a fraction of the input voltage that can be easily measured with an analog oscilloscope or digitizer. The divider itself must have adequate insulation and physical dimensions large enough to withstand the full applied voltage, but must also have the wide bandwidth necessary to scale microsecond or submicrosecond high-voltage transients with minimal distortion. Additionally, the voltage recorder must have sufficient resolution to measure the fast waveforms. Kerr cells, on the other hand, are electro-optic transducers whose optical transmission properties depend upon the applied voltage. They are inherently fast because their response is limited primarily by molecular reorientation times of the Kerr liquid, which are subnanosecond [6]. Additional restrictions on the temporal response of the Kerr cell measurement system are imposed by the bandwidth limitations of the photodetector used to measure the transmitted light. The improvements in measurement techniques using both dividers and Kerr cells enable the determination of divider ratios of test dividers with less than 0.4 % expanded relative uncertainties. The uncertainty is established using a coverage factor of *k* = 2 and is thus a 2 standard deviation estimate [7, 8]. The definition of expanded measurement uncertainties is found in Refs. [7] and [8], and will be discussed in subsequent sections of this paper. The level of uncertainty of 0.4 % is less than the requirements of the standards applicable to high-voltage impulse measurements by nearly an order of magnitude for ordinary laboratory dividers, and by over a factor of two for reference measurement systems [1, 5]. The next section of this paper presents a description of the reference voltage divider system developed at NIST followed by a discussion of the design and operation of Kerr electro-optic measurement systems. The paper concludes with a discussion of the comparative measurement techniques used for determination of the ratios of pulse voltage dividers.

## 2. NIST Impulse Voltage Divider Measurement System

### 2.1 Resistive Divider Measurement Uncertainties

**Fig. 1:**
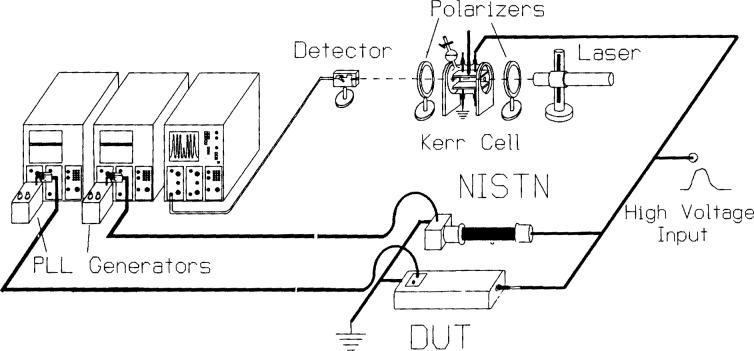
Resistive divider and Kerr electro-optic high-voltage impulse measurement systems. The basic system consists of a light source, crossed polarizers, Kerr cell, photodetector, amplifier, and oscilloscope. (DUT is the divider under test.)

**Fig. 2:**
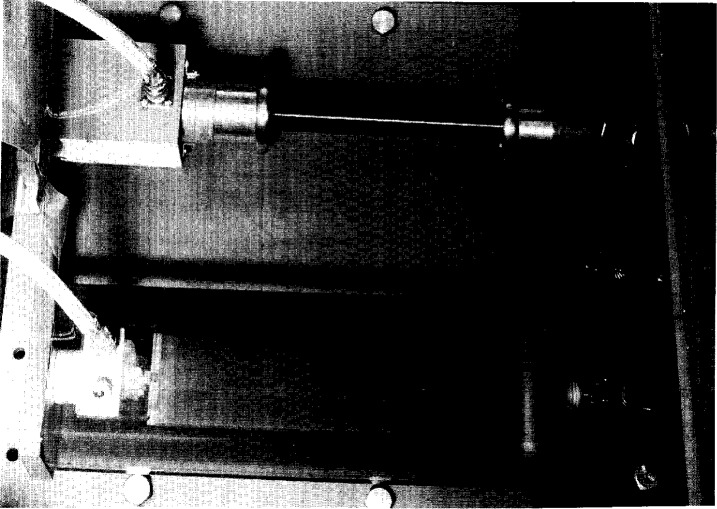
Photo of NISTN and divider under test (DUT).

The purpose of an impulse voltage divider measuring system is to provide a means of reducing the high-voltage signal to levels which are compatible with data recording equipment. In the ideal case, the voltage divider linearly scales the high voltage *U* with a fixed ratio *D*_R_:

$$U=D_{R}V,$$ (1)

where, since the output voltage *V* is measured at the voltage recorder, *D*_R_ is an overall ratio for the system consisting of a voltage divider, signal cables, and terminator, as shown in Fig. 1. The stray capacitances and residual inductances associated with resistive dividers, however, cause them to have a ratio that is frequency-dependent. For accurate impulse voltage measurements it is necessary to have a divider ratio that is relatively constant throughout the frequency range of interest. The NIST reference divider is designed to have a constant ratio over this frequency range, which for measurements of impulse voltages having characteristic times of the order of microseconds is from dc to 10^7^ Hz [9]. The reference voltage divider designated NISTN shown in Fig. 2 has been designed and constructed at NIST. NISTN is a compact device that is placed in a large oil-filled tank containing the output pulse transformer of the high-voltage generating circuit. The NISTN divider is placed adjacent to the divider under test (DUT) as shown in the figure.

The voltage range and divider ratio of the NISTN divider were selected to be comparable to the dividers to be tested. NISTN has been designed with a nominal resistance of 10^4^ Ω, a nominal divider ratio of 5250:1, and covers the voltage range from 10 kV to 300 kV. The output of the divider is connected to a 50 Ω coaxial cable approximately 5 m in length that is terminated at the voltage recorder end by switchable attenuator. The attenuator terminates the cable in its characteristic impedance and optionally provides an additional factor of 10:1 attenuation.

The standard uncertainty, (i.e., a 1 standard deviation estimate) of the high voltage measured by the reference divider NISTN, δ(*U*), is found by applying the law of propagation of uncertainty to Eq. (1):

$$\delta^{2}(U)=V^{2}\delta^{2}(D_{R})+D_{R}^{2}\delta^{2}(V),$$ (2)

where δ(*D*_R_) and δ(*V*) are the standard uncertainties of the ratio *D*_R_ and output voltage *V*, respectively. The relative standard uncertainty in the high voltage, δ_r_(*U*) is

$$\delta_{r}(U)={\left[\delta_{r}^{2}(D_{R})+\delta_{r}^{2}(V)\right]}^{1/2},$$ (3)

where $\delta_{r}^{2}(U)\equiv \delta^{2}(U)/{\left(U\right)}^{2}$, $\delta_{r}^{2}(D_{R})\equiv \delta^{2}(D_{R})/D_{R}^{2}$, and $\delta_{r}^{2}(V)\equiv \delta^{2}(V)/V^{2}$. The law of propagation of uncertainties can be found in the Appendix A of this paper and in Ref. [8]. To minimize the uncertainty in the measured high voltage, the uncertainties in both the reference divider ratio and the measured divider output voltage, δ_r_(*D*_R_) and δ_r_(*V*), respectively, must be minimized; the major sources of these uncertainties are given in Table 1. The overall ratio *D*_R_ is determined by the impedances of the signal cables, signal and divider grounds, and attenuator, in addition to those of the divider itself. The uncertainty in *D*_R_ is primarily associated with uncertainties in the measurement system impedances while the uncertainty in the output voltage is primarily associated with uncertainties in the scale factors of the voltage recorder. The other factors listed in Table 1 are minimized through careful design and shielding of the measurement system.

**Fig. 3:**
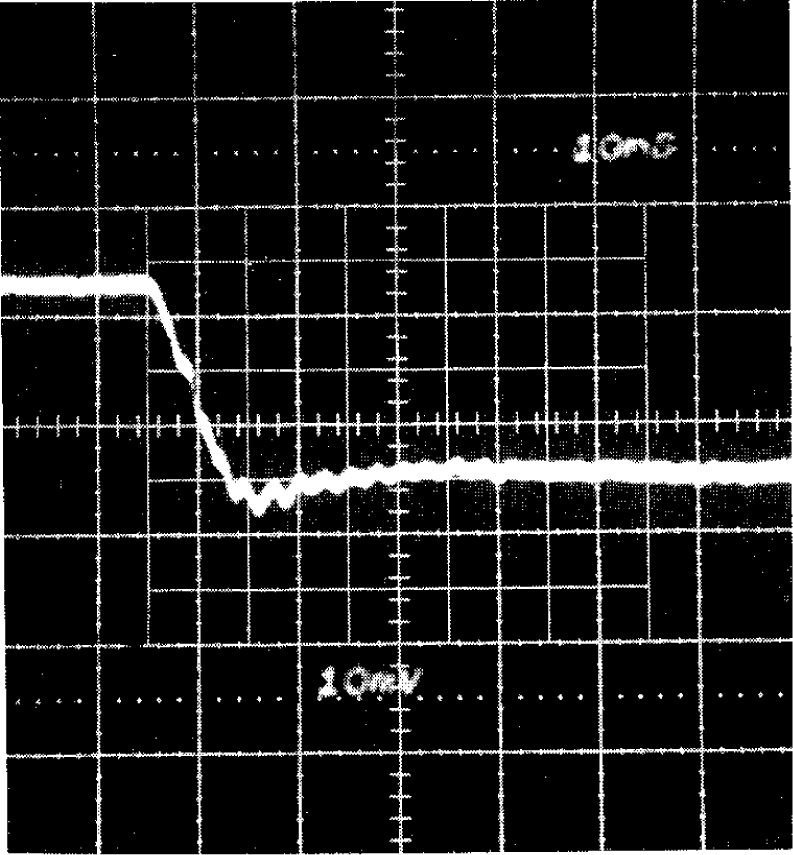
Setup response of NISTN voltage divider.

The NIST reference voltage divider is of the resistive type, i.e., the device’s impedance is primarily resistive. NISTN does, however, have stray capacitances and residual inductances that cannot be entirely eliminated. The divider has a capacitive shield to grade the voltage along its high-voltage arm to eliminate partial discharges at the high-voltage input and to reduce pickup of unwanted radiated and coupled signals that distort the scaling of the high voltage. These intrinsic capacitances and inductances would cause a frequency-dependent divider ratio *D*_R_, but for the NISTN divider *D*_R_ deviates significantly from its low-frequency value only at frequencies outside the range for which the impulse waveforms to be measured have significant components, i.e., >10^7^ Hz. This is experimentally verified through measurement of the low-voltage step response of the divider measurement system by applying a dc voltage of approximately 200 V to the divider and then connecting it to ground through a fast switch such as a mercury-wetted relay. The step response technique is also described in the IEEE and IEC standards on high-voltage impulse measurements [1, 5]. The step response of the NISTN divider is shown in Fig. 3. The response time as defined in IEEE Standard 4 [1] was calculated to be less than 15 ns [10] and thus qualifies for accurate measurement of standard lightning impulses having characteristic times of microseconds. The step response reaches steady state after about 90 ns.

In addition to the capacitance ring at the high-voltage input used to grade the voltage along the length of the high-voltage arm, NISTN has a small ring at the bottom of the high-voltage arm. Unwanted signals, such as those radiated from the high-voltage switch that are coupled to the divider near its high-voltage input, are attenuated and their distortions of the low voltage output signal from the divider are small. If they are coupled directly to the bottom of the high-voltage arm they are attenuated less and their distorting effects are greater, but by placing a small capacitive ring at the bottom, this area of the high-voltage arm is shielded from undesirable radiated signals. The same is true of unwanted signals coupled directly from the high-voltage input to the lower windings of the high-voltage arm; pickup from external sources is thereby minimized. A test is performed to ensure that this pickup is negligible. The high-voltage input to the voltage divider is disconnected from the impulse generator and connected to ground, the generator is energized at a test voltage level, and the output of the voltage divider is measured. The pickup is found to be substantially less than 0.1 % of what the normal output of the divider would be at that voltage level. The penalty paid for including a capacitance ring at the bottom of the high-voltage arm is an increase in the response time and a frequency-dependent divider ratio. This is not a significant problem since the capacitance added by the ring is not excessive and the measured response time of less than 15 ns is still small enough for the divider to measure microsecond impulses accurately.

Another source of uncertainty in impulse measurements is poor ground connections between the voltage divider, which is located near the high-voltage generator, and the voltage recorder, which is located in a shielded room at a distance of some five meters from the generator. To avoid problems of pickup and voltage drops across the signal cable ground, the cable is run through a braided sheath placed on a copper sheet 15.2 cm wide connected to the signal ground at both the divider and oscilloscope ends. The effective dc ground impedance is measured to be less than 30 mΩ, making this source of uncertainty in the divider ratio negligible.

### 2.2 Pulse Level Line (PLL) Method

The second term in Eq. (3), $\delta_{r}^{2}(V)$, is associated with the voltage measurement. Through the use of a special technique known as the pulse level line (PLL) method, conventional analog storage oscilloscopes which generally have specified relative uncertainties of the order of 1 % can have relative standard uncertainties in the measured output voltage peak reduced to less than 0.1 % [11]. Variations of a basic method called the “slideback” measurement technique, which uses a voltage reference or references, are used to ensure or improve the accuracy of a peak impulse voltage measurement. In the slideback technique, an offset voltage is applied to a storage oscilloscope input and the peak of the impulse is measured relative to a known dc voltage level that is applied to the oscilloscope input after the impulse voltage is measured. Because the dc level provides an independent voltage reference, it is possible to use a more sensitive vertical scale on the oscilloscope than would otherwise be required if the ground line was used as reference. Similarly, in another commonly-used method called the “level line” measurement technique, the peak measurement is based upon two dc level lines which are selected to be slightly greater and slightly smaller in amplitude than the peak of the impulse.

**Fig. 4:**
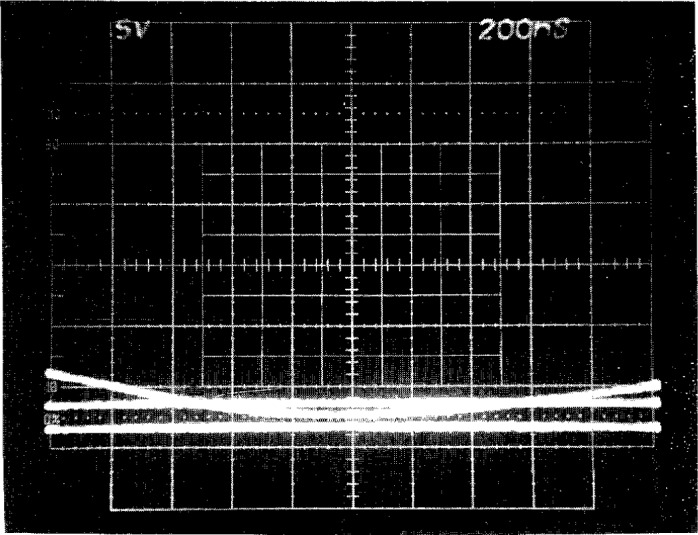
Photographic record of the storage oscilloscope screen. The three traces shown are: the impulse voltage measured from a precision high-voltage divider and two reference level lines.

The accuracy of these methods relies upon the assumption that the oscilloscope amplifier circuits have the same response to the voltage impulse as they do to the dc stimulus. If there are slight differences in how the oscilloscope amplifier responds to an impulse versus dc, then the dc level line and slideback methods do not provide the best accuracy for the peak voltage measurement. We have therefore devised a method to provide the application of reference voltage levels to the oscilloscope in the form of fast-rising voltage steps. This method more closely simulates the conditions under which impulse voltages are measured and avoids possible problems associated with differences between the dc and the impulse measurement amplifier responses. Thus the amplifier is stimulated by the calibration level lines in a manner similar to the impulse to be measured. The voltage at the oscilloscope input does not instantaneously rise to the level line voltage when it is applied, but rather rises as (1−e^−^*^t/τ^*), where *τ* is the charging time constant of the oscilloscope and is less than 100 ns. The pulse level line comparisons are made only at times longer than 8*τ* when the level line is within 0.034 % of the final level. A photographic record of the storage oscilloscope screen with the PLL traces is shown in Fig. 4.

The relative standard uncertainty in the output voltage peak δ_r_(*V*_p_) using the PLL method is estimated to be approximately 0.06 %, as shown in Appendix A. The PLL technique has been verified by measuring a standard voltage step maintained by the Electricity Division at NIST [12]. The average of four measurements of the 5 V step using the PLL technique was within 0.02 % of its calibrated value.

Components of the standard uncertainty in the NISTN divider ratio due to the effects listed in Table 1 are minimized through the design and shielding considerations described above. The relative expanded uncertainty (coverage factor of *k* = 2 and thus a 2 standard deviation estimate) in the test divider ratio is less than 0.4 %, which is based upon a relative standard uncertainty in the reference divider ratio δ_r_(*D*_R_) that is estimated to be less than 0.2 % through comparison with Kerr cell measurement systems, as described in Appendix A. Using the estimate for δ_r_(*V*_p_) of 0.06 % and the estimate for δ_r_(*D*_R_), the relative expanded relative uncertainty in the peak voltage measured by the test divider is found from Eq. (3) to be less than 0.4 %, using a coverage factor of two. This is more than a factor of two smaller than the requirement of 1 % uncertainty in peak voltage measurement for reference measurement systems as defined by international standards.

Kerr cell measurement systems are far more complicated than those based on dividers. They therefore require greater care and are usually limited to use in controlled laboratory environments. They have been used at NIST and elsewhere for many years for the measurement of high electric fields and high voltages because their excellent measurement uncertainty at high-voltage can exceed that of voltage dividers [13, 14, 15, 16, 17]. Kerr cell systems and the techniques used with them for high-voltage impulse measurements are described in the next section.

## 3. Kerr Electro-Optic Impulse Voltage Measurement Systems

### 3.1 Theory of Operation of Kerr Cells

Kerr cells are electro-optic transducers whose optical properties change when high voltage is applied to them. A typical Kerr cell and major components of the optical system are shown in Fig. 1. The system consists of a light source, a Kerr cell with polarizers at its input and output, a light detector for optical to electrical conversion, and a voltage recorder to measure the detector output. The Kerr cell itself is essentially a parallel plate capacitor connected to the high-voltage circuit at the point where the voltage is to be measured; the electric field between the plates is uniform. The cell contains a Kerr liquid such as nitrobenzene (C_6_H_5_NO_2_) which becomes birefringent when high voltage is applied to the electrodes of the cell: the electric field between them induces a difference between the index of refraction for light linearly polarized in the direction parallel to the field, *n_z_*, and light polarized perpendicular to it, *n_y_*. This induced difference in the refractive indices is proportional to the square of the electric field between the electrodes, *E*^2^:

$$\Delta n=n_{z}-n_{y}=BE^{2}.$$ (4)

**Fig. 5:**
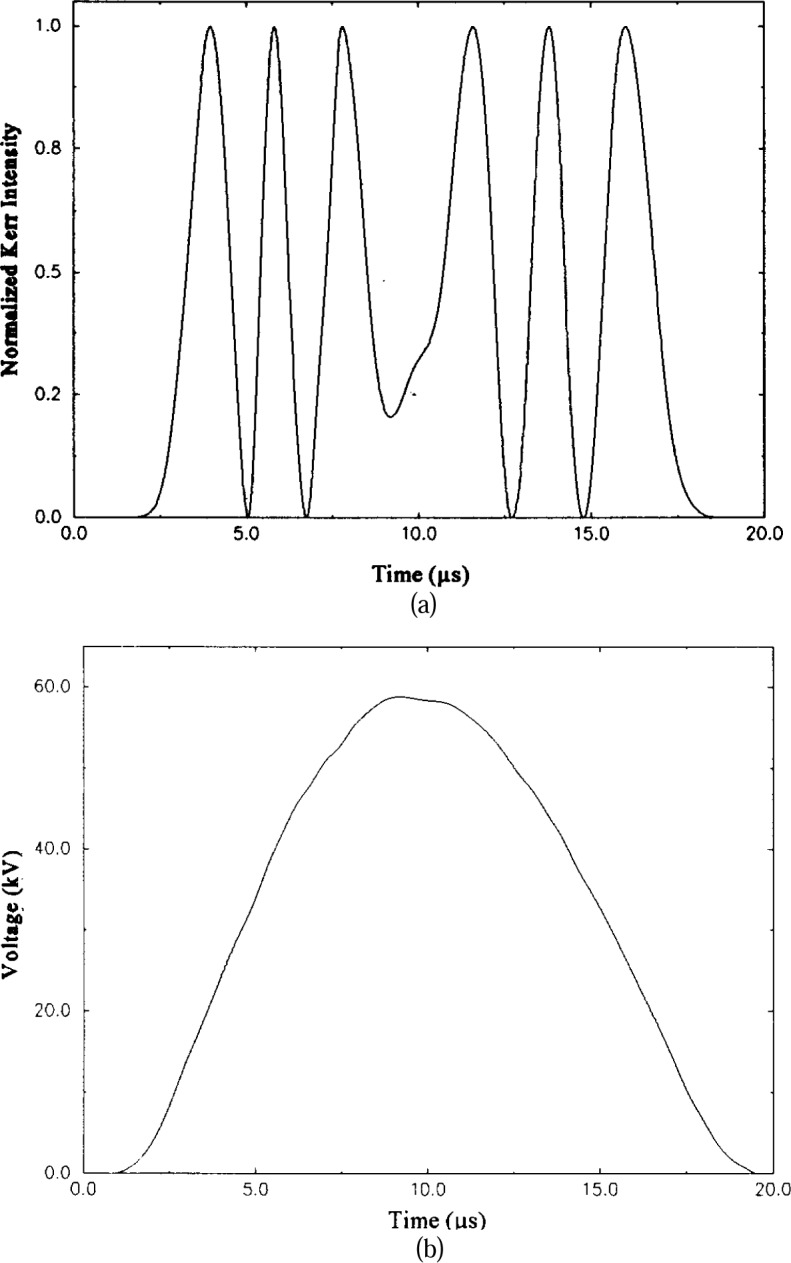
Kerr Measurement system output signal. a) the sinusoidal variation in light intensity produced by the Kerr cell and polarizers as measured by the photodetector; b) the applied voltage as measured by a precision high-voltage divider.

In this equation, *B* is known as the Kerr coefficient and has both a temperature and wavelength dependence. Nitrobenzene has the largest known Kerr coefficient among dielectric liquids having fast response characteristics. As illustrated in Fig. 1, the incident light beam passes through a polarizer that has its optical axis oriented –45° to the direction of the applied electric field between the plates so that at the entrance to the Kerr cell the light is linearly polarized with components of equal magnitude and phase in the *y* and *z* directions. The induced birefringence results in a phase delay between these components of the incident beam as they pass through the cell so that at the output of the cell the polarization is changed from linear to elliptical. This change in polarization is measured using an analyzer at the cell output that is oriented perpendicularly to that of the polarizer at the input. With no applied voltage, very little of the incident beam reaches the photodetector. Figure 5 shows the measured intensity of the beam at the Kerr cell output that is oriented perpendicularly to that of the polarizer at the input. With no applied voltage, very little of the incident beam reaches the photodetector. Figure 5 shows the measured intensity of the beam at the Kerr cell output as a high-voltage impulse is applied, with the applied voltage shown for comparison. As the voltage increases, the induced change in the polarization of the beam causes more and more of the beam to be passed by the analyzer until the transmission is maximized. Further increases in the applied voltage causes less light to be transmitted until minimum transmission is reached again. As the voltage is increased even further, the light transmission increases again and the cycle is repeated.

The number of oscillations in light intensity is determined by the amplitude of the applied voltage and the cell constant, a parameter that at constant temperature is fixed by the cell geometry and Kerr electro-optic coefficient *B* of the liquid. The relation of the measured output light intensity *I* to the applied voltage *U* for an ideal Kerr measurement system is given by [18]

$$I/I_{m}=\sin^{2}[(\pi /2){\left(U/U_{m}\right)}^{2}].$$ (5)

*I*_m_ is the light intensity at maximum transmission. The cell constant *U*_m_ is defined as

$$U_{m}=d/{\left(2Bl\right)}^{1/2},$$ (6)

where *d* is the electrode spacing and *l* is the electrode length. The term “cell constant” has an historical basis and in fact is not strictly constant because it is a function of the Kerr coefficient *B*, which changes with temperature and wavelength. The high-voltage impulse measurements are made with monochromatic light and temperature corrections to *U*_m_ are made using the measured temperature dependence of *B* for nitrobenzene [19]. The dependence of *B* on temperature *T* is given by

$$B(T)=\alpha_{0}+\alpha_{1}T^{-1}+\alpha_{2}T^{-2},$$ (7)

where the parameters *α*_0_, *α*_1_, and *α*_2_ that produce the best fit to measured data are given in Ref. [19] and also the Appendix to this paper. The relationship between the cell constant at temperature *T*_2_ and the cell constant at temperature *T*_1_ is from Eq. (6)

$$U_{m2}=U_{m1}{\left[B(T_{1})/B(T_{2})\right]}^{1/2}.$$ (8)

where *U*_m1_ ≡ *U*_m_(*T*_1_) and *U*_m2_ ≡ *U*_m_(*T*_2_). In general, the Kerr cell constant is calibrated at temperature *T*_1_ and corrected using Eqs. (7) and (8) to the temperature *T*_2_, which is the cell temperature at the time of test divider calibration.

Equation (6) is derived by assuming that the applied electric field encountered by the light beam is uniform and contained entirely between the plate electrodes. Since there are always nonuniform fringing fields at the edges of the electrodes, the Kerr cell constant *U*_m_ will differ from that calculated using Eq. (6). However, the edge effects can be accounted for by replacing the electrode length *l* with an effective electrode length *l*' in Eq. (6).

Each half-cycle of the Kerr output waveform is historically called a “fringe” because it is the result of either constructive or destructive interference of the orthogonal components of the output light beam, as in an interference fringe pattern. The fringe number *n* is defined as the square of the ratio of the voltage applied to the cell to the cell constant:

$$n\equiv {\left(U/U_{m}\right)}^{2}.$$ (9)

The applied voltage at any time *t* can then be reconstructed from the Kerr waveform by substituting Eq. (9) into Eq. (5) and solving for *n* to get

$$n(t)=\{\begin{array}{c} N+\frac{2}{\pi }\sin^{-1}\sqrt{\frac{I(t)}{I_{m}}},\;N\;\text{even},\\ N+1-\frac{2}{\pi }\sin^{-1}\sqrt{\frac{I(t)}{I_{m}}},\;N\;\text{odd}, \end{array}$$ (10)

which can be summarized as

$$\begin{array}{l} n(t)=N+2\left(\frac{N}{2}-\mathrm{int}\left(\frac{N}{2}\right)\right)\\ +{\left(-1\right)}^{N}\frac{2}{\pi }\sin^{-1}\sqrt{\frac{I(t)}{I_{m}}}. \end{array}$$ (11)

Here, *N* = int(*n*) is the integer part of *n* and int(*N*/2) is the integer part of *N*/2. The voltage is found from Eq. (9) to be

$$U=n^{1/2}U_{m}.$$ (12)

The high voltage input can be calculated from the Kerr cell output waveform using Eq. (12).

### 3.2 Sources of Uncertainty in Kerr Cell Measurements

The accuracy of the Kerr measurement depends upon several system characteristics listed in Table 2. Additionally, the measurements are sensitive to other effects, particularly the presence of electric charges within the liquid which distort the normally uniform electric field between the electrodes. In general, the Kerr cell response time is in the 10 ns range or less and is more than adequate for the measurement of microsecond transients. The same is true of the bandwidths of voltage recorders such as analog oscilloscopes and digitizers, which can exceed 10^8^ Hz.

The peak voltage *U* of an impulse may be found from Eq. (12) by counting the number of fringes (cycles) *n* of the waveform of the type shown in Fig. 5, and using the Kerr cell constant *U*_m2_, which is the Kerr cell constant calculated from *U*_m1_ by applying the correction for temperature. The relative standard uncertainty in the peak voltage measurement, $\delta_{r}^{2}(U)$, is found from Eq. (12) to be (see Appendix A)

$$\delta_{r}(U)={\left[\delta_{r}^{2}(n)/4+\delta_{r}^{2}(U_{m})\right]}^{1/2},$$ (13)

where as before δ_r_(*U*) ≡ δ(*U*)/*U*, and where δ_r_(*n*) ≡ δ(*n*)/*n*, and δ_r_(*U*_m_) ≡ δ(*U*_m_)/*U*_m_ are the relative standard uncertainties in the fringe number and Kerr cell constant, respectively. Equation (13) illustrates a useful property of Kerr cells for the measurement of high-voltage impulses: If the standard uncertainty in the fringe number δ(*n*) is only a fraction of a fringe and is independent of fringe number, the relative standard uncertainty δ_r_(*n*) decreases with increasing *n* (i.e., as the applied voltage increases). In the limit of very large fringe number the relative standard uncertainty δ_r_(*U*) depends solely upon the cell constant uncertainty. Thus, the uncertainty at higher voltages may be less than at lower voltages. This upper bound in the standard uncertainty in the fringe number can be understood by examining Eq. (10), which shows that *n* comprises two components, an integer fringe number *N*, and a fractional component. The uncertainty in the fringe number then has two components, namely the uncertainties in the integer and fractional parts. The uncertainty in the integer part *N* is negligible because the large difference in the measured peak voltage determined with the Kerr cell system and either the reference or test divider would be immediately apparent if *N* was miscounted, even if by only one integer fringe. The uncertainty in the fractional part is less than 0.006, as estimated in the Appendix A, and therefore δ(*n*) is bounded.

The uncertainty in the measured fringe number arises from those sources listed in Table 2 that affect *I* and *I*_m_. Even if these sources produce a standard uncertainty in the fringe number as large as 0.01, the relative standard uncertainty is reduced to the order of 0.1 % for voltage levels producing more than ten fringes.

The Kerr voltage measurement system used at NIST for testing of compact voltage dividers uses an intensity-stabilized helium-neon laser as a light source which has negligible variation in the intensity *I*_m_ over the measurement time window of less than 15 μs. The effects of positioning the optical elements in the measurement system are two-fold: first, misalignment of the polarizers introduces a constant phase shift between the beam components in the *y* and *z* directions in addition to that produced by the induced birefringence; and second, misalignment of the beam results in a change in effective path length *l*′ which changes the cell constant according to Eq. (6). The dependence of effective length on position of the beam has been derived by Thacher [20]:

$$\begin{array}{c} l^{\prime }=l\{1+(d/l\pi \}\\ {}[1+0.5\ln(2\pi z/d)/\sin(2\pi z/d)]\}. \end{array}$$ (14)

In this equation, the electrode spacing is *d*, the physical electrode length is *l*, and the vertical displacement from the center of the parallel, horizontally-mounted electrodes is *z*. If the error in position *z* is 10 % (*z*/*d* = 0.01), then the resultant change in *l*′ is less than 0.05 % for *d* = 0.635 cm and *l* =15.24 cm. With proper care in alignment, significant errors in the effective cell constant are avoided.

The dynamic response of the Kerr cell is a potential source of error in the effective cell constant, but this is limited by the dipolar relaxation time *τ*, which characterizes the dependence of dielectric constant on the frequency of the applied electric field, known as the dielectric dispersion. Measurements of the dielectric constant of nitrobenzene, however, show it to be frequency-independent from dc to 10^8^ Hz [21]. The errors for the pulses used in divider tests, which have minimal frequency components above a few megahertz, are also believed to be negligible.

**Fig. 6:**
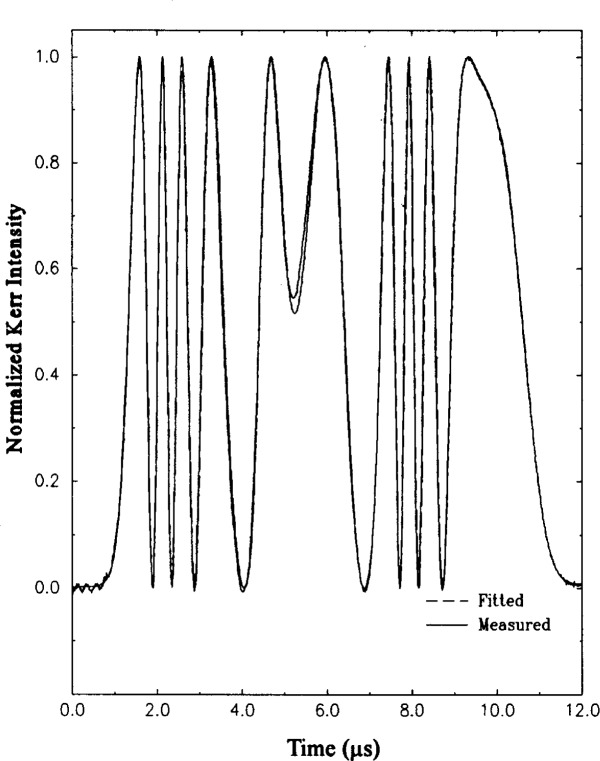
Ideal and actual Kerr measurement system output waveforms. The actual measured Kerr system waveform with the ideal waveform calculated from Eq. (1) superimposed.

To minimize the uncertainties and errors of Kerr cell measurements, the linearity of the opto-electrical photodetector must be calibrated and maintained to within 1 % or less. The absence of significant nonlinearity in the Kerr measurement is seen in Fig. 6, which shows the measured output of the Kerr cell system. The curve superimposed upon the measured curve is that calculated from the applied voltage measured simultaneously by the reference voltage divider and calculated using Eq. (9). The curves are normalized to emphasize the difference in their temporal responses. Although the fitted waveform does not match the measured waveform at the points corresponding to the peak voltage in Fig. 6, the relative difference in the fringe numbers calculated from the two waveforms is less than 0.02 %. The operating conditions of the photodetectors have been optimized to have nonlinearities of less than 0.1 % [11].

The oscilloscopes and digital recorders used in the calibrations have 3 dB bandwidths between 100 MHz and 400 MHz, which are adequate for the measurement of approximately 100 fringes with the pulses used for the testing of dividers. There are practical upper limits to the number of fringes that are usable when analog storage oscilloscopes are used to record the Kerr traces. The practical limit with analog storage devices is the resolution of the measured Kerr fringes as determined by the width of the trace and the “bloom” of the storage screen. This limit has been found to be approximately 100 fringes.

In addition to the uncertainties in the measured fringes, the major source of the uncertainties in Kerr measurements is the value of the Kerr cell constant *U*_m1_, calibrated at temperature *T*_1_, used to calculate the peak voltage from the Kerr trace. This value is calibrated through comparison of pulse voltage measurements with a second reference voltage divider. Uncertainties in the cell constant correction are introduced through the uncertainty in the measurement of the cell temperature, which is less than 0.1 °C. This uncertainty in cell constant could be reduced if the Kerr cell calibration could be performed using ac or dc voltages since the ratios of steady-state voltage dividers such as those used for dc or ac voltages are known with much lower uncertainty than impulse dividers. The difficulty in performing the Kerr cell calibrations with ac or dc is that significant electric charge appears in the liquid when the voltage is applied for times greater than 10^−4^ s that appreciably distorts the electric field in the electrode gap. The field distortion modifies the relationship between the field in the center of the cell and the voltage on the electrodes so that Eq. (5) is no longer valid. The cell must therefore be calibrated using impulse voltages, where the effects of charges in the liquid are insignificant for times typically less than 100 μs [18].

NIST maintains a pair of Kerr cells with overlapping ranges. Cell B has a characteristic Kerr cell constant of 6.4 kV at 21.2 °C and Cell C has one of 46.8 kV at 24.2 °C. When pulses having peak voltages of 50 kV to 60 kV are applied, the output of Cell C has only one or two fringes while that of Cell B has nearly 100 fringes. The large fringe number from Cell B provides much lower uncertainty in the peak voltages measured in this range than Cell C. At higher voltages, the Kerr measurements with Cell C have smaller uncertainties than at the lower voltages levels.

The NISTN divider together with the Kerr cell systems have been used in complementary fashion to calibrate other compact impulse dividers using comparative measurements. These techniques are described in the next section.

## 4. Comparative Measurement Techniques

Impulse voltage measurement systems invariably introduce some distortion due to inadequate bandwidth, voltage coefficient, and other factors. This distortion may be either negligible or totally unacceptable, depending on the allowable uncertainties associated with the particular measurement requirement. According to IEEE and IEC standards [1, 5], a system which is used to measure standard lightning impulses should have an uncertainty of less than 3 % in peak voltage measurements. For reference measurement systems the standards require an uncertainty of 1 % in peak voltage measurement. The standards also recommend that the dynamic behavior of the measurement system can be evaluated by using parameters obtained from the step response, but a more reliable and simplified method prescribed by the standard is based on simultaneous measurements of a high-voltage impulse by an independent reference system and the system under test. As a first step, international comparative measurements were made in four national laboratories and the relative differences among them were reported [22]. Investigations have also been made of the interactions between two systems configured for simultaneous measurements and of methods for minimizing these interactions [9].

**Fig. 7:**
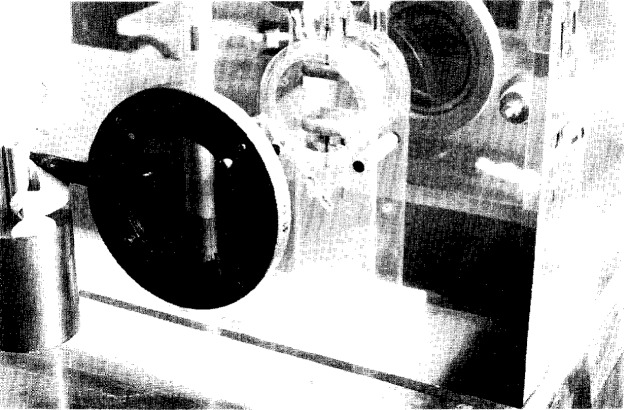
Kerr cell and high-voltage impulse generator. The Kerr cell is immersed in an oil bath to provide temperature stability. The oil-filled tank beneath the Kerr cell contains the high-voltage transformer and both the reference divider and divider under test.

Determinations of the voltage divider ratio of a test divider are performed at NIST by making simultaneous measurements with a well-characterized measurement system—either the reference voltage divider, Kerr cell, or both. The peak voltage measured by the reference system is used with the output voltage of the test divider to determine the unknown divider ratio *D*_R_ according to Eq. (1). For compact dividers the comparison is made between the test divider and the reference divider, NISTN, in which the test and reference dividers are placed side-by-side under oil, close to the output of the impulse voltage generator, and connected to the generator with a very low-impedance conductor. The Kerr system is also placed close to both dividers and connected to the impulse generator via a low-impedance conductor. The Kerr cell system, seen in Fig. 7, is immersed in a mineral oil bath located on top of the impulse generator. The oil bath prevents flashover around the cell and partial discharges on the surface of the cell when high-voltage pulses are applied, and also provides temperature stability. Temperature measurements are periodically taken for correction of the cell constant.

The high-voltage pulse generator consists of a pulse-forming network (PFN) charged to a high dc voltage that is switched into a pulse transformer. The pulse shape can be modified somewhat by simply removing or adding inductors in the PFN circuit. The waveform that is typically used in the testing of impulse dividers is Gaussian-shaped, having a full width at half maximum of approximately 8 μs and a total duration of less than 15 μs.

Preliminary measurements of the test divider are made before placing the divider into the test system. The resistive components measured include the resistances of the high-voltage arm, low voltage arm, cable center conductor, sheath, and terminator. The overall voltage ratio is then calculated from the equivalent circuit and the measured resistance values. After the divider is installed in the test system, the dc voltage ratio is found by applying a range of dc voltages between 25 V and 250 V to the test divider and simultaneously measuring the input and output with precision digital multimeters. The pickup test described in Sec. 2.1 is performed by grounding the input to the test divider in situ and energizing the impulse generator. The final low voltage test on the divider that is made is the step response measurement where a dc voltage is applied to the divider and then rapidly switched to ground via a mercury-wetted relay. The output of the divider is measured to ensure that the response time of the divider is not excessively long. The test divider step response is similar to the NISTN step response shown in Fig. 3.

To evaluate the impulse voltage ratio of a resistive divider under test the following procedure is used: When the high-voltage impulse is applied, the oscilloscopes are simultaneously triggered to capture the output waveforms of the NIST reference divider, the divider under test, and the Kerr cell, or some combination of the three. Two dual-channel analog storage oscilloscopes having bandwidths of 100 MHz are used to capture the three waveforms. Photographs of the stored waveforms are taken within a few seconds and position measurements of the level lines, peak voltages, and Kerr waveform parameters are made with a caliper mounted on a platen which secures the photographs. A computer program has been written to perform the calculations for the peak output voltage from the measurements from the Kerr cell and from the dividers. The heights of the pulse level lines and of the divider output voltage peak are entered with the level line voltages for each divider and the reference divider ratio. The peak output voltages are then calculated according to the pulse level line technique. The heights of the baseline intensity *I*_0_, intensity maximum *I*_m_, and intensity *I* corresponding to the voltage peak are measured from the Kerr waveform and entered along with the Kerr cell temperature and Kerr cell constant *U*_m_ at the calibration temperature *T*_1_. The program calculates the peak input voltages applied to the NIST divider and Kerr cell system and provides a hardcopy output. The ratio of the test divider is determined using both NISTN and the Kerr cell as reference and displays the difference of the two to ensure the consistency of the results. The unknown divider ratio is found in this way over the desired voltage range, typically 10 kV to 300 kV.

The expanded uncertainty in the test divider ratio over this voltage range determined by the comparison is estimated to be less than 0.4 % using a coverage factor of *k* = 2. The test pulse voltage dividers are used as reference dividers by other laboratories such as Sandia National Laboratories, which has developed an automated calibration system for reference/test divider comparisons [23]. The divider ratio of the reference divider measurement system NISTN determined by calculation from the measurements of its component resistances and from low voltage measurements at dc agree to within 0.1 %. The expanded relative uncertainty of the reference divider ratio estimated through comparisons with reference Kerr cell systems using high-voltage impulses is less than 0.2 %. Efforts continue at NIST to further reduce this uncertainty. Techniques are being developed for the characterization of the Kerr cell constant *U*_m_ through comparison with a reference divider using digital rather than analog recorders. The digitized data permit the comparison to be made over the entire impulse voltage waveform, instead of only at the voltage peak as done with the analog oscilloscopes. Curve fitting techniques have been used with Eq. (6) to find the cell constant that minimizes the error between the fitted and calculated curves using the voltage waveform determined by the reference divider [24].

NIST also has the capability of testing the ratio of free-standing voltage dividers. The accuracy in impulse measurements at high voltages that is possible with compact dividers immersed directly in the tank housing the output pulse transformer of the high-voltage generator is greater than that achievable with free-standing dividers, but uncertainties in the ratio determination of less than 1 % may be achievable. The free-standing reference divider NIST4 is similar in design to the NISTN divider: The high-voltage arms are similar, consisting of resistive wire counterwound on a glass ceramic substrate and surrounded by insulating oil; the low-voltage side is an array of parallel discrete resistors [10]. NIST has a 500 kV Marx-type impulse generator used to produce standard lightning impulses, which have rise-times of approximately 1.5 μs and fall to half the peak value in 50 μs. The Marx impulse voltage generator produces more radiated noise than the pulse-forming network (PFN)-type and therefore unwanted signals of significant amplitude may be coupled to the freestanding dividers. A 600 kV PFN-type generator is presently being installed and tested. Its use should reduce the uncertainties due to pickup of extraneous signals in impulse measurements using free-standing voltage dividers.

## 5. Conclusion

The NIST test facility enables the determination of the ratio of compact pulse voltage dividers with expanded relative uncertainties of less than 0.4 % using a coverage factor of *k* = 2. The ratio determinations are made through comparative measurements with both a reference voltage divider and Kerr electro-optic measurement system. NIST continues efforts to improve high impulse voltage measurements with free-standing voltage dividers to reduce uncertainties even further below the 1 % level to support the international standards governing high-voltage test techniques used by industrial laboratories, which require the verification that uncertainties in impulse voltage measurements not exceed 1 % as determined through direct intercomparison with reference measurement systems traceable to national standards laboratories [1, 5].

## Figures and Tables

**Table 1:** Possible sources of voltage divider measurement uncertainties

Effect	Parameter affected
Nonconstant scale factor over frequency range of interest	*D*_R_
Heating of windings (divider ratio dependent on temperature)	*D*_R_
Voltage coefficient (divider ratio dependent on voltage)	*D*_R_
Poor circuit grounding	*D*_R_
Pickup of radiated and coupled signals	*V*
Voltage recorder signal distortion	*V*

**Table 2:** Possible sources of uncertainty in high-voltage impulse measurements with Kerr cells

Type of effect	Description Parameters	affected
Optical	Light source intensity stability	*I*, *I*_m_
	Spectral purity of light	*U*_m_
	Beam width	*U*_m_
	Alignment of beam with central axis of cell	*U*_m_
	Alignment of polarizers	*I*, *I*_m_
	Beam bending due to polarizers	*I*, *I*_m_
	Internal reflections within the cell	*U*_m_
	Presence of additional birefringent elements	*I*, *I*_m_
Electro-optical	Purity of Kerr liquid	*U*_m_
	Presence of significant electric charge (space charge)	*U*_m_
	Temperature variations in the Kerr liquid	*U*_m_
	Electric field uniformity between cell electrodes	*U*_m_
	Photodetector dynamic response and linearity	*I*, *I*_m_
	Dimensional changes in the cell due to temperature changes	*U*_m_
Electrical	Voltage recorder signal distortion	*I*, *I*_m_
	Dynamic response of Kerr cell	*U*_m_

**Table 3:** Typical values of divider measurement parameters

Parameter		Value	Equation number
Peak input voltage	*U*_P_, *U*_K_, *U*_R_	10 kV to 300 kV	1, 15, 16a, 16b, 22, 23, 24a, 24b, 38

Voltage divider ratio	*D*_R_	5250	1, 2, 16a, 19a, 22, 23, 40
	*D*_T_	5100 to 5300	15, 17a, 17b

Peak output voltage	*V*_R_, *V*_R_, *V*_T_	1.9 V to 58.8 V	1, 2, 15, 16, 17a, 17b, 19a, 20, 22, 23, 24a, 39, 42

Height measurements (from photographs)	*h*_1_	7.369 cm	20, 21
	*h*_P_	7.569 cm	20, 21
	*h*_2_	7.656 cm	20, 21

Reference dc voltage measurements	*V*_1_, *V*_2_	1.86 V to 61.8 V	20, 21

**Table 4:** Typical values of Kerr cell measurement parameters

	Parameter	Value	Equation number
	*N*	2 to 80	10, 11, 28
	*n*	2.40 to 80.25	10, 11, 16b, 17b, 22, 23, 24a, 28, 39, 42
	*U*_m1_	6386 V, 46770 V	8, 25, 27a, 40
	*U*_m2_	6449 V, 46390 V	8, 16b, 17b, 22, 23, 24a, 25, 42, 43
	*B*_1_	3.27 × 10^–12^ m/V^2^,	8, 25, 27a, 34, 39
Kerr cell mesurement parameters	*B*_2_	3.22 × 10^−12^ m/V^2^,	8, 25, 27a, 34, 39
	*α*_0_	6.128 × 10^−12^ m/V^2^	26, 34, 35, 36, 37
	*α*_1_	−5.287 × 10^−9^ K^2^	26, 34, 35, 36, 37
	*α*_2_	1.310 × 10^−6^ K^2^ m/V^2^	26, 34, 35, 36, 37
	*T*_1_	294.9 K to 297.4 K	34, 35, 36, 37
	*T*_2_	296.1 K to 296.5 K	34, 35, 36, 37
	Δ*I_n_*/Δ*I*_m_	0.03 to 1.00	28, 29, 30
Height measurements (from photographs)	*h*_0_	0.216 cm	30, 31, 32, 33
	*h*_n_	2.289 cm to 6.472 cm	30, 31, 32, 33
	*h*_m_	6.48 cm	30, 31, 32, 33

**Table 5:** Standard uncertainties of comparison parameters

Standard uncertainty	Values	Type of uncertainty	Equation number
δ(*h*)	2.5 × 10^−5^ m	A	21, 31, 32, 33
δ(*V*)	0.0002 V to 0.0059 V	B	21
δ(*V*_P_), δ(*V*_R_), δ(*V*_T_)	0.001 V to 0.035 V	B	19a, 24a, 42
δ(*D*_R_)	8.9	B	2, 19a, 24a
δ(*D*_T_)	9.7 to 10.1	B	19a, 42
δ(*n*)	0.003 to 0.05	B	24a, 29, 33, 42
δ(*U*_m2_)	5.77 V to 41.40 V	B	24a, 27a, 42
δ(*U*_K_ − *U*_R_)	17 V to 510 V	B	24a
δ(*B*_1_/*B*_2_)	0.00179 to 0.00185	B	27a, 35, 36
δ(Δ*I_n_*/Δ*I*_m_)	0.0007 to 0.0008	B	29, 31, 32
δ(*T*)	0.05 K	B	36, 37

**Table 6:** Relative standard uncertainties of comparison parameters

Relative standard uncertainty	Maximum value (%)	Type of uncertainty	Equation number
δ_r_(*V*)	0.01	B	22
δ(*V*_P_), δ(*V*_R_), δ(*V*_T_)	0.06	B	19b, 24b, 38, 41, 43
δ_r_(*D*_R_)	0.17	B	19b, 24b, 38, 41
δ_r_(*D*_T_)	0.19	B	19b, 41, 43
δ_r_(*n*)	0.02	B	13, 24b, 38, 43
δ_r_(*U*_m2_)	0.05	B	24b, 27b, 43
δ_r_(*U*_K_ − *U*_R_)	0.15	A	24b, 38
δ_r_(*B*_1_/*B*_2_)	0.09	B	27b, 37, 38

## 6. Appendix A. Uncertainty Considerations

The uncertainties of the voltage ratio of the divider under test (DUT) are dependent upon the uncertainties associated with the measurement of the output voltages of the DUT, a reference measurement system which is either a reference voltage divider or a photodetector that is used with a Kerr cell, and the uncertainties associated with the relationships of these reference output voltages to their input voltages. The input voltages to the DUT and the reference measurement system are the same since they are connected in parallel. The expanded uncertainty in the voltage ratio of the test divider is estimated beginning with the simple relationship between the input and output voltages for a resistive voltage divider:

$$D_{T}=U_{p}/V_{T}.$$ (15)

Here *D*_T_ is the ratio of the DUT, *U*_p_ is the peak input voltage, and *V*_T_ is the measured peak output voltage of the DUT. The input voltage is an impulse waveform that monotonically increases to the peak voltage *U*_p_ and then monotonically decreases, as shown in Fig. 5b. *U*_p_ is found either from simultaneous measurement with a reference divider having ratio *D*_R_ or a Kerr cell measurement system having temperature-corrected cell constant *U*_m2_ as

$$U_{p}=\{\begin{array}{cc} D_{R}V_{R}\equiv U_{R} & \left(16a\right)\\ n^{1/2}U_{m2}\equiv U_{K}, & \left(16b\right) \end{array}$$

which gives for *D*_T_

$$D_{T}=\{\begin{array}{cc} \left(D_{R}V_{R}\right)/V_{T}=f\left(D_{R},V_{R},V_{T}\right) & \left(17a\right)\\ n^{1/2}U_{m2}/V_{T}=f\left(n,U_{m2},V_{T}\right) & \left(17b\right) \end{array}$$

where the peak output voltage of the reference divider is *V*_R_ and the fringe number at the voltage peak is *n*. The standard uncertainty in the unknown divider ratio *D*_T_ is estimated by applying to Eq. (15) the law of propagation of uncertainty, which in general form is [8]

$$\begin{array}{c} \delta_{c}^{2}\left(y\right)=\sum_{i=1}^{N}{\left(\partial f/\partial x_{i}\right)}^{2}\delta^{2}\left(x_{i}\right)\\ +2\sum_{i=1}^{N-1}\sum_{j=i+1}^{N}\left(\partial f/\partial x_{i}\right)\left(\partial f/\partial x_{j}\right)\delta \left(x_{i},x_{j}\right). \end{array}$$ (18)

It defines the relationship between the combined standard uncertainty in the output quantity *y*, *u*_c_(*y*), and the quantities δ(*x_i_*), which are the standard uncertainties of the input quantities *x*_i_. The second term in the above equation sums to zero over many measurements if the input quantities are uncorrelated, which is true in the two cases described by Eqs. (17a) and (17b).

### 6.1 Uncertainties for Divider-Divider Comparisons

Applying the law of propagation of uncertainty to Eqs. (17a), the uncertainty in the DUT ratio is found from comparison with the reference divider output to be

$$\begin{array}{c} \delta^{2}\left(D_{T}\right)={\left(V_{R}/V_{T}\right)}^{2}\cdot \delta^{2}\left(D_{R}\right)+{\left(D_{R}/V_{T}\right)}^{2}\cdot \delta^{2}\left(V_{R}\right)\\ +{\left(D_{R}V_{R}/V_{T}^{2}\right)}^{2}\cdot \delta^{2}\left(V_{T}\right), \end{array}$$ (19a)

or when written in relative form

$$\delta_{r}^{2}\left(D_{T}\right)=\delta_{r}^{2}\left(D_{R}\right)+\delta_{r}^{2}\left(V_{R}\right)+\delta_{r}^{2}\left(V_{T}\right),$$ (19b)

where $\delta_{r}^{2}\left(D_{T}\right)\equiv \delta^{2}\left(D_{T}\right)/D_{T}^{2}$, $\delta_{r}^{2}\left(D_{R}\right)\equiv \delta^{2}\left(D_{R}\right)/D_{R}^{2}$, $\delta_{r}^{2}\left(V_{R}\right)\equiv \delta^{2}\left(V_{R}\right)/V_{R}^{2}$, and $\delta_{r}^{2}\left(V_{T}\right)\equiv \delta^{2}\left(V_{T}\right)/V_{T}^{2}$. These equations show that the uncertainty in the unknown divider ratio depends on the uncertainties in the reference divider ratio, the reference divider output voltage, and the test divider output voltage.

The relative uncertainties in output voltages δ_r_(*V*_R_) and δ_r_(*V*_T_) in Eqs. (19a) and (19b) are the same in magnitude since the same technique and equipment are used in both measurements. If the divider ratios of the test and reference dividers are close, then their output voltages are approximately the same. The output voltages are measured using the pulse level line (PLL) technique described in Sec. 2.2 and their associated uncertainties are estimated by applying the law of propagation of uncertainty to the defining equation for the PLL method, as shown in Sec. 6.1.1.

One approach that has been used to estimate δ(*D*_R_) is to determine the ratio at a low dc voltage, where both the input and output voltages can be measured with a precision digital multimeter, and then perform a voltage linearity check by measuring the peak output of the divider as a function of the charging voltage of the high-voltage generator [1]. Alternatively, the divider ratio can be calculated from the measured component resistances together with the high-voltage linearity check [1]. Because of the instabilities and large uncertainties in the dc high-voltage supply and the charging voltage meter, nonlinearities in the high-voltage generator, corona, switching energy dissipation, and other effects, these approaches cannot be used to reliably estimate the reference divider uncertainty. In general, the divider ratio uncertainties are much smaller than those of the generator and meter that are used to check voltage linearity, so the overall uncertainties are dominated by components other than that of the divider ratio. However, δ(*D*_R_) can be indirectly estimated by taking the difference between the amplitudes of the voltage peaks that are measured simultaneously using a Kerr cell (*U*_K_) and the reference voltage divider (*U*_R_), as will be shown in Sec. 6.1.2.

#### 6.1.1 Divider Output Voltage Measurement Uncertainties

The uncertainties in the peak output voltages for the test and reference dividers, δ(*V*_T_) and δ(*V*_R_), have the same magnitude for the reasons cited above. To estimate these uncertainties the general relationship between the measured peak output voltage *V*_p_ and the reference voltage levels for the PLL technique are used [11]:

$$V_{p}=V_{1}+\left(V_{2}-V_{1}\right)\left(h_{p}-h_{1}\right)/\left(h_{2}-h_{1}\right),$$ (20)

where *h*_p_ is the measured height of *V*_p_, *h*_1_ is the measured height of pulse level line 1, *h*_2_ is the measured height of pulse level line 2, *V*_1_ is reference voltage 1, and *V*_2_ is reference voltage 2. The standard uncertainty δ(*V*_p_) is found by applying the law of propagation of uncertainty to Eq. (20):

$$\begin{array}{l} \delta^{2}(V_{p})=\{[{\left(h_{2}-h_{p}\right)}^{2}+{\left(h_{p}-h_{1}\right)}^{2}]/{\left(h_{2}-h_{1}\right)}^{2}\}\delta^{2}(V)\\ \;+\{[{\left(V_{2}-V_{1}\right)}^{2}/{\left(h_{2}-h_{1}\right)}^{4}][{\left(h_{2}-h_{1}\right)}^{2}\\ \;+{\left(h_{2}-h_{p}\right)}^{2}+{\left(h_{p}-h_{1}\right)}^{2}]\}\delta^{2}(h). \end{array}$$ (21)

Terms containing δ(*V*_1_) and δ(*V*_2_) have been combined since these uncertainties have the same magnitude, which is designated δ(*V*) = |δ(*V*_1_)| = |δ(*V*_2_)|. Similarly, terms containing δ(*h*_1_), δ(*h*_p_), and δ(*h*_2_) have also been combined in Eq. (21) using δ(*h*) ≡ |δ(*h*_1_)| = |δ(*h*_P_)| = |δ(*h*_2_)|.

The standard uncertainty in the height measurements δ(*h*) is estimated to be 0.0025 cm. The standard uncertainties in the voltage measurements δ(*V*) is taken from the manufacturer’s specifications to be 0.01 % of *V*. Using the values given in Table 3 for the heights and reference voltages, the uncertainty in *V*_p_ is estimated from Eq. (22) to be between 0.001 V and 0.033 V over the 10 kV to 300 kV range for input voltages. The relative uncertainty in *V*_p_, δ_r_(*V*_p_) ≡ δ(*V*_p_)/*V*_p_, is less than 0.06 %. Typical heights are *h*_1_ = 7.369 cm, *h*_2_ = 7.569 cm, and *h*_p_ = 7.656 cm, and the reference voltages *V*_1_ and *V*_2_ usually differ by less than 4 % of their mean.

#### 6.1.2 Reference Divider Ratio Uncertainty

The uncertainty of the reference voltage divider ratio δ(*D*_R_) is evaluated indirectly through a series of simultaneous measurements made with the divider and a Kerr cell. The difference in the peak voltage simultaneously measured by the Kerr cell and reference divider is

$$\Delta U_{\text{KR}}\equiv U_{K}-U_{R}=n^{1/2}U_{m2}-D_{R}V_{R},$$ (22)

where *U*_m2_ is the temperature-corrected Kerr cell constant and *n* is the fringe number described in Sec. 3. Solving Eq. (22) for the divider ratio *D*_R_ one obtains

$$D_{R}=\left[n^{1/2}U_{m2}-\left(U_{K}-U_{R}\right)\right]/V_{R}.$$ (23)

The standard uncertainty for the reference divider ratio is

$$\begin{array}{l} \delta (D_{R})=\{[U_{m2}^{2}/(4nV_{R}^{2})]\delta^{2}(n)+(n/V_{R}^{2})\delta^{2}(U_{m2})\\ +\delta^{2}(U_{K}-U_{R})/V_{R}^{2}+(U_{R}^{2}/V_{R}^{4})\delta^{2}(V_{R}){\}}^{1/2}, \end{array}$$ (24a)

and the relative standard uncertainty for this divider ratio is

$$\begin{array}{c} \delta_{r}(D_{R})=(U_{K}/U_{R})\{[(\delta_{r}^{2}(n)/4+\delta_{r}^{2}(U_{m2})]\\ +\delta_{r}^{2}(U_{K}-U_{R})+\delta_{r}^{2}(V_{R}){\}}^{1/2}, \end{array}$$ (24b)

with $\delta_{r}^{2}\left(U_{K}-U_{R}\right)\equiv \delta^{2}\left(U_{K}-U_{R}\right)/U_{R}^{2}$, $\delta_{r}^{2}\left(n\right)\equiv \delta^{2}\left(n\right)/n^{2}$, and $\delta_{r}^{2}\left(U_{m2}\right)\equiv \delta_{r}^{2}\left(U_{m2}\right)/U_{m2}^{2}$. Note that the relative standard uncertainty δ_r_(*U*_K_ − *U*_R_) in the difference of the two peak voltage measurements is not defined as the uncertainty of the difference divided by the difference, but rather as the uncertainty of the difference divided by the peak input voltage *U*_R_ determined by the divider.

Equation (24b) shows that the relative standard uncertainty of the reference divider ratio can be estimated from estimates of the relative uncertainties of the Kerr measurement parameters δ_r_(*n*) and δ_r_(*U*_m2_), the difference of the peak voltages δ_r_(*U*_K_ − *U*_R_), and the reference divider output voltage δ_r_(*V*_R_). The uncertainty in the output voltage was estimated in the previous section and the Kerr measurement parameter uncertainties are estimated in the following section. The uncertainty of the difference of peak input voltages is estimated from measurement data. The expanded uncertainty for the test divider is estimated in Sec. 6.1.4.

#### 6.1.3 Kerr Cell Measurement Uncertainties

The determination of the test divider ratio is performed with the Kerr cell at temperature *T*_2_ which in general is different from the temperature *T*_1_ at which the calibration was performed, but can be calculated using [19]:

$$U_{m2}=U_{m1}{\left(B_{1}/B_{2}\right)}^{1/2},$$ (25)

where *B*_1_ and *B*_2_ are the Kerr electro-optic coefficients at temperatures *T*_1_ and *T*_2_, respectively, as discussed in Sec. 3.1. The temperature dependence of the Kerr coefficient of nitrobenzene was measured by Hebner and Misakian, who fit the resultant data to a curve described by [19]

$$B(T)=\alpha_{0}+\alpha_{1}T^{-1}+\alpha_{2}T^{-2}.$$ (26)

From Eq. (26) the standard uncertainty of *U*_m2_ is

$$\delta^{2}(U_{m2})=((B_{2}/B_{1})\cdot U_{m1}^{2}/4)\delta^{2}(B_{1}/B_{2}),$$ (27a)

which can be rewritten in relative terms as

$$\delta_{r}^{2}(U_{m2})=\delta_{r}^{2}(B_{1}/B_{2})/4,$$ (27b)

using $\delta_{r}^{2}\left(U_{m2}\right)\equiv \delta^{2}\left(U_{m2}\right)/U_{m2}^{2}$ and $\delta_{r}^{2}\left(B_{1}/B_{2}\right)\equiv \delta^{2}\left(B_{1}/B_{2}\right)/{\left(B_{1}/B_{2}\right)}^{2}$. Because in the series of measurements used for the statistical evaluation of δ_r_(*U*_K_ − *U*_R_), the calculation of *U*_K_ is made with either one of the two constants *U*_m1_ given in Table 4, there is no component of uncertainty in *U*_m1_ due to random effects in this evaluation, i.e., *U*_m1_ is constant and has no statistical variations in these tests. The uncertainties in *U*_m1_ due to systematic effects are taken into account in the estimation of the combined measurement uncertainty of *U*_K_, but these are believed to be small, as discussed in the next section.

The expression for fringe number *n* in terms of the intensity *I*_n_ corresponding to *n* and the maximum and baseline intensities *I*_m_ and *I*_0_ is

$$n=\{\begin{array}{c} N+\frac{2}{\pi }\sin^{-1}\sqrt{\frac{\Delta I_{m}}{\Delta I_{m}}},\;N\;\text{even},\\ N+1-\frac{2}{\pi }\sin^{-1}\sqrt{\frac{\Delta I_{n}}{\Delta I_{m}}},\;N\;\text{odd}, \end{array}$$ (28)

where Δ*I_n_* = *I_n_* − *I*_0_ and Δ*I*_m_ = *I*_m_ − *I*_0_. The uncertainty in *n* is found from Eq. (28) to be

$$\delta^{2}(n)=\{(1/\pi^{2})[1/(\Delta I_{n}/\Delta I_{m})-{\left(\Delta I_{n}/\Delta I_{m}\right)}^{2}]\}\delta^{2}(\Delta I_{n}\Delta I_{m}).$$ (29)

The intensities *I*_n_, *I*_m_, and *I*_0_ are determined from a photograph of the output of the photodetector displayed on the storage oscilloscope. They are measured in terms of the heights on the photograph just as the output voltages *V*_R_ are measured. The ratio is found as

$$\begin{array}{l} \Delta I_{n}\equiv I_{n}-I_{0}=k(h_{n}-h_{0}),\\ \Delta I_{m}\equiv I_{m}-I_{0}=k(h_{m}-h_{0}), \end{array}$$

and

$$\Delta I_{n}/\Delta I_{m}=(I_{n}-I_{0})/(I_{m}-I_{0})=(h_{n}-h_{0})/(h_{m}-h_{0}),$$ (30)

where *h_n_*, *h*_0_, and *h*_m_ are the measured heights of the intensity traces corresponding to fringe number *n*, the baseline intensity level, and the maximum intensity level, respectively. The constant *k* includes the electro-optic efficiency of the photodetector, the trans-impedance of the amplifier circuit, and the oscilloscope scale factors, but because a ratio is used in Eq. (30), the factor *k* cancels since it is reasonable to assume that it is the same for *I*_n_, *I*_m_, and *I*_0_. Thus the uncertainty in the ratio Δ*I_n_/*Δ*I*_m_ depends only on the uncertainty in the height measurements

$$\begin{array}{l} \delta^{2}\left(\Delta I_{n}/\Delta I_{m}\right)={\left(h_{m}-h_{0}\right)}^{-2}\delta^{2}\left(h_{n}\right)\\ +[\left(h_{n}-h_{m}\right)/{\left(h_{m}-h_{0}\right)}^{2}{]}^{2}\delta^{2}\left(h_{0}\right)\\ +[-\left(h_{n}-h_{0}\right)/{\left(h_{m}-h_{0}\right)}^{2}{]}^{2}\delta^{2}\left(h_{m}\right). \end{array}$$ (31)

As for the PLL technique, the standard uncertainties for all of the height measurements have the same magnitude, designated δ(*h*), so that the terms in Eq. (31) can be combined to give

$$\begin{array}{l} \delta^{2}\left(\Delta I_{n}/\Delta I_{m}\right)=\{[{\left(h_{m}-h_{0}\right)}^{2}{\left(h_{n}-h_{m}\right)}^{2}\\ +{\left(h_{0}-h_{n}\right)}^{2}]/{\left(h_{m}-h_{0}\right)}^{4}\}\delta^{2}\left(h\right), \end{array}$$ (32)

Substituting Eq. (32) into Eq. (29) yields

$$\begin{array}{c} \delta^{2}\left(n\right)=\{[{\left(h_{m}-h_{0}\right)}^{2}+{\left(h_{n}-h_{m}\right)}^{2}\\ +{\left(h_{0}-h_{n}\right)}^{2}]/[\pi^{2}\times \left(1-[(h_{n}-h_{0}\right)/\left(h_{m}-h_{0}\right)])\\ \times [\left(h_{n}-h_{0}\right)/\left(h_{m}-h_{0}\right)]\times {\left(h_{m}-h_{0}\right)}^{4})]\}\delta^{2}\left(h\right). \end{array}$$ (33)

The above equation does not contain an uncertainty term for the integer fringe number *N* shown in Eq. (28) because δ(*N*) is zero; an error in counting the integer number of fringes in a Kerr trace would be immediately detected in the large difference *U*_K_ − *U*_R_ that would result. The uncertainty in *n* then depends upon the heights of the intensity traces measured from the oscilloscope photograph which determine the fractional component of *n* and on the uncertainty of the height measurements δ(*h*), but not upon the integer component *N*. For the typical height values given in Table 3 and for the standard uncertainty δ(*h*) of 0.025 cm as used previously in the PLL output voltage calculation in Sec. 2.2, the standard uncertainty in *n* ranges from 0.05 to 0.25, which corresponds to a relative standard uncertainty in *n*, δ_r_(*n*), of less than 0.02 % over the voltage range used.

The standard uncertainty in the Kerr cell constant at the divider calibration temperature *T*_2_ in Eq. (27b) depends on the relative standard uncertainty in the ratio of the Kerr coefficients δ_r_(*B*_1_/*B*_2_). To determine the relative standard uncertainty δ_r_(*U*_m2_), Eq. (26) is used to obtain the ratio:

$$B_{1}/B_{2}=(\alpha_{0}+\alpha_{1}T_{1}^{-1}+\alpha_{2}T_{1}^{-2})/(\alpha_{0}+\alpha_{1}T_{2}^{-1}+\alpha_{2}T_{2}^{-2}).$$ (34)

The standard uncertainty is then

$$\begin{array}{l} \delta^{2}\left(B_{1}/B_{2}\right)={\left[(\alpha_{1}T_{1}^{-2}+2\alpha_{2}T_{1}^{-3})/B_{2}\right]}^{2}\delta^{2}\left(T_{1}\right)\\ +{\left[\left(B_{1}/B_{2}^{2}\right)\left(\alpha_{1}T_{2}^{-2}+2\alpha_{2}T_{2}^{-3}\right)\right]}^{2}\delta^{2}\left(T_{2}\right). \end{array}$$ (35)

The same values of *α*_0_, *α*_1_, and *α*_2_ are used for measurements at all Kerr cell temperatures and therefore there is no random component of uncertainty. The systematic component of uncertainty is believed to be negligible because of the excellent agreement between the simultaneous Kerr cell and reference voltage divider measurements made for cell temperatures between 293.6 K to 297.3 K. Any error in the values of *α*_0_, *α*_1_, and *α*_2_ used would result in either a monotonic increase or decrease in the difference between the peak voltage determined with the two measurement systems as the Kerr cell temperature changed, but only random changes in this difference were seen; no systematic trends in the data were observed. The uncertainties of the temperature coefficients are therefore negligible.

The magnitudes of the standard uncertainty of the measured temperatures *T*_1_ and *T*_2_ are the same and designated δ(*T*), so Eq. (35) reduces to

$$\begin{array}{l} \delta^{2}\left(B_{1}/B_{2}\right)=\{{\left[\left(\alpha_{1}T_{1}^{-2}+2\alpha_{2}T_{1}^{-3}\right)/B_{2}\right]}^{2}\\ +{\left[\left(B_{1}/B_{2}^{2}\right)\left(\alpha_{1}T_{2}^{-2}+2\alpha_{2}T_{2}^{-3}\right)\right]}^{2}\}\delta^{2}\left(T\right) \end{array}$$ (36)

or in relative form

$$\begin{array}{l} \delta_{r}^{2}\left(B_{1}/B_{2}\right)=\{{\left[\left(\alpha_{1}T_{1}^{-2}+2\alpha_{2}T_{1}^{-3}\right)/B_{1}\right]}^{2}\\ +{\left[\left(\alpha_{1}T_{2}^{-2}+2\alpha_{2}T_{2}^{-3}\right)/B_{2}\right]}^{2}\}\delta^{2}\left(T\right), \end{array}$$ (37)

with δ_r_(*B*_1_/*B*_2_) ≡ δ(*B*_1_/*B*_2_)/(*B*_1_/*B*_2_).

#### 6.1.4 Test Divider Ratio Uncertainty

The relative standard uncertainty for the reference divider found by substituting Eq. (27b) into Eq. (24b) is

$$\begin{array}{c} \delta_{r}(D_{R})=(U_{K}/U_{R})\{[\delta_{r}^{2}(n)+\delta_{r}^{2}(B_{1}/B_{2})]/4\\ +\delta_{r}^{2}(U_{K}-U_{R})+\delta_{r}^{2}(V_{R}){\}}^{1/2}. \end{array}$$ (38)

An estimate for the relative standard uncertainty of the difference of the peak voltages measured by the Kerr cell and reference divider, δ_r_(*U*_K_ − *U*_R_)/*U*_R_, of 0.15 % has been obtained from the sample standard deviation of a series of measurements covering the voltage range from 10 kV to 300 kV. Using this estimate and the values for the other parameters and their uncertainties listed in Tables 3 through 6, δ_r_(*D*_R_) is calculated to be at most 0.17 %.

The estimate for δ_r_(*D*_R_) is made using components of uncertainty due to random effects and does not include components due to systematic effects, which are believed to be negligible. This conclusion is based on the evaluation of the difference of the peak voltages (*U*_K_ – *U*_R_)/*U*_R_, which for this series of measurements has a mean of less than 0.1 %. The difference in peak voltage measurements given in Eq. (22) can be recast using Eq. (25) as

$$\Delta U_{\text{KR}}\equiv {\left[n(B_{1}/B_{2})\right]}^{1/2}\Delta U_{m1}-\Delta D_{R}V_{R},$$ (39)

where Δ*U*_m1_ and Δ*D*_R_ are the systematic errors of the cell constant and reference divider ratio, respectively, and *U*_R_ = *D*_R_*V*_R_ = [*n*(*B*_1_/*B*_2_)]^1/2^ *U*_m1_. Using *n*^1/2^ = *U*_p_/*U*_m2_ = *U*_p_/[*U*_m1_(*B*_1_/*B*_2_)^1/2^], Eq. (39) becomes

$$\Delta U_{\text{KR},r}=\Delta U_{m1}/U_{m1}-\Delta D_{R}/D_{R},$$ (40)

with Δ*U*_KR,r_ ≡ Δ*U*_KR_/*U*_P_. It is possible that the relative errors in the cell constant and divider ratio are both large and that only their difference in Eq. (40) is small, but this is unlikely; it would mean that there would be a significant difference between the low voltage divider ratio calculated from the component resistances, or the low voltage dc ratio, and the ratio at the high voltages used in this comparison. Such a difference may arise from heating and voltage effects, but it is unlikely that these effects would result in a constant divider ratio over the high voltage range covered here. A constant difference in Eq. (40) for a divider ratio that changes with voltage would mean that the cell constant would have to change equally, but this would indicate either a change in the physical dimensions of the cell or a change in the Kerr liquid temperature. No temperature changes have been measured during the comparison testing that would indicate such a phenomenon is actually occurring. Additionally, a large change in the divider ratio from low voltage to high voltage would be seen in the Kerr waveform fit to the measured output voltage, shown in Fig. 6, for which the constant low-voltage divider ratio was used. If the divider ratio were voltage dependent, the fit at the start of the trace (the lower voltage values) would be good, but the fit near the peak (the highest voltage values) would be poor, which is not the case as is clearly seen from the figure. Although the fitted waveform does not match the measured waveform at the points corresponding to the peak voltage in Fig. 6, the relative difference in the fringe numbers calculated from the two waveforms is less than 0.02 %. Simultaneous measurements with other pulse voltage dividers show similar excellent agreement. It is therefore concluded that the relative errors in both the divider ratio and the cell constant are insignificant.

The estimate of the combined standard uncertainty for the unknown divider ratio from Eq. (19b) is found to be

$$\delta_{r}(D_{T})={\left[\delta_{r}^{2}(D_{R})+2\delta_{r}^{2}(V_{p})\right]}^{1/2},$$ (41)

where the magnitude of δ_r_(*V*_T_) and δ_r_(*V*_R_) are the same and designated δ_r_(*V*_p_). With the parameter and uncertainty values from Tables 3 through 6, δ_r_(*D*_T_) is calculated to be 0.19 %. Using a coverage factor of *k* = 2 the expanded relative uncertainty in the test divider ratio is 0.38 %.

### 6.2 Uncertainties for Divider—Kerr Cell Comparison

Applying the law of propagation of uncertainties to Eq. (17b), the uncertainty in the test divider ratio found through comparison with a Kerr cell is

$$\begin{array}{c} \delta^{2}(D_{T})=[U_{m}^{2}/(4n\cdot V_{T})]\delta^{2}(n)+(n/V_{T})\delta^{2}(U_{m2})\\ +(n\cdot U_{m2}^{2}/V_{T}^{4})\delta^{2}(V_{T}), \end{array}$$ (42)

or in relative form

$$\delta_{r}^{2}(D_{T})=\delta_{r}^{2}(n)/4+\delta_{r}^{2}(U_{m2})+\delta_{r}^{2}(V_{T}).$$ (43)

The uncertainty in the temperature-corrected cell constant *U*_m2_ calculated in the previous sections only accounts for components arising from random effects. Systematic effects are also considered to be negligible for the reasons given in the previous sections. Using the values for δ_r_(*n*) and δ_r_(*U*_m2_) in Table 6 based on the derivations given in the previous sections and the parameter values in Table 3, the relative standard uncertainty δ_r_(*D*_T_) in the test divider ratio is 0.11 %. This gives a total relative expanded uncertainty of less than 0.22 % using a coverage factor of *k* = 2.
